# The course of depression in late life: a longitudinal perspective

**DOI:** 10.1017/S204579602000058X

**Published:** 2020-07-29

**Authors:** Alejandro de la Torre-Luque, Jose Luis Ayuso-Mateos

**Affiliations:** 1 Department of Legal Medicine, Psychiatry and Pathology, Universidad Complutense de Madrid, Spain; 2 Centre for Biomedical Research in Mental Health (CIBERSAM), Spain; 3 Department of Psychiatry, Universidad Autónoma de Madrid, Spain

**Keywords:** Depression trajectories, late-life depression, loneliness, persistent depression, symptom approach

## Abstract

Depression in old age deserves special attention in view of the fact of progressive population ageing, because of the way in which depression and risk factors interact in this period of life and the particularly negative impact of late-life depression on health and quality of life. This editorial aims to provide some insight into longitudinal aspects of depression in old age. Depression may follow varying trajectories (e.g. episode emergence, recurrence) across the lifespan. Late-life depression is not an exception. A symptom-based approach is presented as an appropriate research method to study the predictors and course of affective syndromes in old age. Findings from our studies on depressive symptom trajectories in old age revealed that participants with a course of unremitting elevated symptoms showed the highest levels of loneliness across the trajectory groups and that participants with subclinical symptoms also showed higher levels of loneliness than their counterparts with a minimal-symptom course trajectory. This highlights the need to address loneliness as a way of dealing with depression in old age.

Depression can be conceptualised as a dynamic disease from a lifelong standpoint (Van De Leemput *et al*., 2014; Hosenfeld *et al*., 2015). Depression seems to evolve dynamically across the lifespan in response to internal features (genetic liability and biological processes) and external factors (environmental demands and resources: protective and risk factor influences). Traditionally, two approaches have been followed to study depression dynamics across the lifespan. The diagnosis-based approach consists of taking depression as a syndrome on a binary nature (i.e. presence or absence of a syndrome). Diagnosis-based definitions of depression rely on medical conceptualisations of depressive disorders. From this approach, the course of depression may be depicted by periodical transitions between discernible states (or statuses). Each status would be related to a differential impact on daily living as well as being moderated by concrete external and internal factors. Richards (2011) distinguished between six discernible statuses falling over the continuum of depression: episode emergence, remission, response, recovery, relapse and recurrence. Additionally, other depression statuses were postulated, such as the subclinical episode, persistent depression and intermittent depression, as they show some distinctive features and are relatively prevalent among the general population. For instance, a prevalence rate of almost 5% is observed for unremitting courses of depression episode of at least 2 years duration (Murphy and Byrne, 2012). Between 25 and 40% of patients showing a major depression episode may experience episode repetition (Richards, 2011; Steenland *et al*., 2012).

Another way to study depression from a longitudinal point of view is by adopting a symptom-based approach. Under this perspective, the rigid conceptualization of full-blown disorder as a unit of analysis is relaxed and the focus is set on (any of) its signs or manifestations (symptoms). This standpoint matches with current conceptual frameworks, such as the Research Domain Criteria (RDoC) Initiative, which go beyond traditional diagnostic systems of mental disorders (Cuthbert and Insel, 2013). Addressing depression from its initial stages (e.g. minimum number of symptoms needed to generate distress) may provide new insights into pathophysiological underpinnings which may encourage tailored initiatives to tackle depression (e.g. preventive actions). Moreover, mounting evidence has highlighted the negative consequences of depression from its earliest stages (subclinical depression) as risk factors for psychiatric comorbidity and increased mortality risk (Balázs *et al*., 2013; Cuijpers *et al*., 2013).

The study of depression dynamics from a symptom-based approach deserves particular interest in old age for several reasons. First, late-life depression is also associated with some serious consequences even when the disorder is not fully manifested (i.e. some key diagnostic criteria are missing) in diagnostic terms (Braam *et al*., 2014; Conde-Sala *et al*., 2019; de la Torre-Luque *et al*., 2019*a*). In this vein, diagnosis-based studies may overlook the actual impact of depression in late life. Second, the clinical presentation of depression is different in old age in comparison to earlier periods in life. Thus, somatic symptoms are more prominent in late-life depression than cognitive and emotional ones, in comparison to depression in young and middle adulthood (Hegeman *et al*., 2012). This makes it less probable for depression to be diagnosed in old age as key symptoms for diagnosis are eminently emotional.

There is evidence for an increasing number of depressive symptoms from middle age to late life, even after controlling for comorbid conditions (e.g. chronic diseases, dementia). Moreover, this pattern seems to be universal and free from culture-specific imperatives (Yu *et al*., 2012; Chui *et al*., 2015; de la Torre-Luque *et al*., 2019*a*). Conversely, studies addressing depression from a diagnosis-based approach have often shown a decreased risk of depression in old age (Han, 2002; Byers *et al*., 2010).

## Trajectories of depression in late life

Several factors should be taken into consideration when studying the longitudinal dynamics of depression in late life. First, the emergence of some risk factors (e.g. presence of chronic disease, progressive sensory impairment) for depression may become particularly prominent in old age (Fiske *et al.*, 2009). Interactions between these factors may put some people at increased risk of either symptom aggravation or full-blown disorder emergence. On the other hand, the influence of some protective factors (e.g. cognitive reserve, psychological resilience) may become buffering agents against depression in this age period. Several trajectories of depressive symptom development may become evident within the overall illness course as a result of the interplay between protective and risk factors and biological predisposition. In fact, many authors have highlighted that a normative (highly-prevalent) course of depressive symptoms has been seen across studies, comprising most of the older adults and characterized by minimal levels of symptoms (Montagnier *et al*., 2014; Musliner *et al*., 2016; de la Torre-Luque *et al*., 2019*a*). Previous research has also identified trajectories of elevated symptoms, as well as trajectories with a steeper increase of symptoms and associated with elevated risks of chronic disease and neuropsychiatric disorder emergence (Chui *et al*., 2015; Singh-Manoux *et al*., 2017).

In a recent study, we described the course of depressive symptoms in a representative sample of more than 8000 older adults from the UK (de la Torre-Luque *et al*., 2019*a*). Analyses were conducted separately for men and women, taking into consideration the differential influence of both biological- and socialisation-related forces on affective processes. As a result, we identified three heterogeneous trajectories of symptoms for both men and women. Most participants (over 77% of men and 68% of women) showed a trajectory of minimal depressive symptoms depicting an increasing pattern over time (never surpassing the cut-off point for clinical meaningfulness). Another class characterized by increasing levels of symptoms (subclinical class) was identified. Levels of symptoms reached the cut-off point of clinical meaningfulness at age 80, on average. Finally, a class (clinical class) comprising about one in ten participants (6% of men and almost 11% of women) was identified. Participants in this class showed a stable pattern of elevated depressive symptoms over time. Symptoms greatly surpassed the cut-off point for clinically relevant symptoms in all the measurement occasions. Additionally, these participants showed very poor quality of life and increased difficulties in activities of daily living. Surprisingly, participants showing the subclinical course of symptoms also exhibited poorer quality of life and higher levels of disability than the individuals depicting a minimal-symptom course.

The study highlighted the importance of considering subclinical states of depression. Furthermore, a course featured by stable and unremitting levels of symptoms was also been uncovered. This finding goes in line with studies showing an increased likelihood of patterns of elevated depression symptoms becoming chronic with age in a substantial proportion of older people (Comijs *et al*., 2015; Musliner *et al*., 2016). These patterns are often more frequent in older women. Additionally, worse outcomes (i.e. higher levels of disability, increased risk of mortality and multimorbidity development) and poor prognosis have been associated with patterns of elevated depression symptoms (or their analogous, diagnostic-based status, persistent depression) in late life (Lenze *et al*., 2005; Luppa *et al*., 2012; Montagnier *et al*., 2014).

Another study conducted by our group depicted the course of depressive symptoms using a multi-state approach. The main aim was to study the dynamics of clinically relevant episodes of depressive symptoms in late life (de la Torre-Luque *et al*., 2019*b*). We used data from the Ageing Trajectories of Health – Longitudinal Opportunities and Synergies (ATHLOS) project (Sanchez-Niubo *et al*., 2019). More concretely, data from more than 40 000 older adults were used, comprising samples from European, American, Asian and Oceanian countries. Follow-up length was 18 years. We found that most participants (over 85%) did not have any symptom episode over the follow-up. Prevalence of episode emergence status was over 5%, and episode persistence was observed in 9.86% of assessment occasions. The episode persistence status became more prevalent with age. In conclusion, it seems that an episode of clinically relevant depressive symptoms may emerge in late life, and the probability of an episode to be repeated/unremitted becomes more evident over time.

## Loneliness in late life

As mentioned before, old age constitutes a period in life that is particularly sensitive in terms of mental disorder dynamics. In that regard, external factors may presumably have a stronger influence on the quality of life and mental health, due to risk factor accumulation (e.g. multimorbidity development, widowhood). Loneliness and social isolation have been put on the spotlight in the last few decades as main contributors of depressive episode emergence and symptom aggravation in old age (Chen *et al.*, 2014; Domènech-Abella *et al*., 2017). Social isolation refers to being physically apart from social interactions. On the other hand, loneliness can be conceptualised as an integrated state of negative feelings and perceptions derived from social isolation (i.e. negative balance between real and ideal quality/quantity of social interactions) (Hawkley and Cacioppo, 2010). Feeling lonely is quite common across the lifespan. Loneliness has been associated with numerous mental and physical conditions, severe disease development (e.g. dementia), metabolic dysregulation and increased mortality risk (Cacioppo *et al*., 2015; Rico-Uribe *et al*., 2018; Lara *et al*., 2019). Furthermore, depression accompanied with loneliness often shows worse prognosis in old age (Holvast *et al*., 2015; Santini *et al*., 2016).

Fortunately, awareness of the devastating effects of loneliness is higher nowadays. Tackling loneliness has become a major priority for some national and international institutions. In fact, some governments have already set up very ambitious initiatives to cope with the effects of loneliness. For instance, the British ministry for loneliness was launched in 2018, developing numerous actions to fight loneliness.

Findings from our group and others have highlighted that loneliness is intimately related to depression, leading to depressive episode emergence and symptom aggravation in old age (Cacioppo *et al.*, 2006; Cacioppo and Cacioppo, 2016; Domènech-Abella *et al*., 2017; de la Torre-Luque *et al*., 2019*a*; de la Torre-Luque *et al*., 2019*b*). Loneliness may trigger some biases in cognitive processes (e.g. selective retrieval of negative memories). These systematic deviations from normative cognitive processing may hinder efficient emotion regulation. Moreover, it may induce some difficulty in emotion processing (e.g. higher accuracy to recognise facial expressions as part of negative emotions), particularly when considering social stimuli (Hawkley and Cacioppo, 2010; Kanai *et al*., 2012). On the other hand, loneliness and perceptions of being socially isolated have been linked with higher levels of some metabolic markers (i.e. leptin) (Häfner *et al*., 2011), endocrine (hypothalamus–pituitary–adrenals axis) dysregulation and increased glucocorticoid resistance (Hawkley and Cacioppo, 2010).

Our study on trajectories of depressive symptoms in old age revealed that participants depicting a course of unremitting elevated symptoms showed the highest levels of loneliness across the trajectory groups (de la Torre-Luque *et al*., 2019*a*). Surprisingly, participants with trajectories of subclinical symptoms of depression also showed higher levels of loneliness than their minimal-symptom trajectory counterparts. Further insight was provided by our study on the dynamics of clinically relevant episodes of depressive symptoms in late life (de la Torre-Luque *et al*., 2019*b*). Loneliness feelings were a highly relevant factor to predict the transition from no episode to symptom episode emergence. Likewise, loneliness was proven to be involved in episode persistence.

## Conclusions

The study of late-life depression has a long-standing tradition in epidemiological and mental health sciences. Likewise, much effort has been made to address the consequences of this health condition. So far, numerous initiatives at both community and individual levels have been developed to minimise the impact of depression and subsequently help people to age well and with good health. However, there are many issues to deal with as depression continues to constitute a substantial disease burden in old age.

Some longitudinal aspects deserve being mentioned when late-life depression is addressed. First, a dual approach which relies on diagnosis as well as on symptoms should be taken. Additionally, adopting a person-centred approach allowing for the identification of individual-specific depression courses could be very helpful. Potential implications in terms of tailoring (personalising clinical assessment and practice) medical decision making and service provision should be highlighted. For instance, the identification of person-specific profiles of interactions between risk factors may help to address highly relevant features such as persistent and intermittent depression. Moreover, findings derived from studying person-specific trajectories of symptoms may help improve precision in predicting treatment response.

Finally, we would like to call for action to tackle loneliness in old age. In this regard, loneliness should constitute a major priority for governments across the globe, as its contribution to depression and other health conditions is highly evident. Further resources (research actions, community-based initiatives, etc.) are needed to fill knowledge gaps on action mechanisms and treatment targets with the final goal of maximising people's opportunities for ageing well.
